# EB1 contributes to microtubule bundling and organization, along with root growth, in *Arabidopsis thaliana*

**DOI:** 10.1242/bio.030510

**Published:** 2018-06-26

**Authors:** Arthur T. Molines, Jessica Marion, Salem Chabout, Laetitia Besse, Jim P. Dompierre, Grégory Mouille, Frédéric M. Coquelle

**Affiliations:** 1 Department of Cell Biology, Institute for Integrative Biology of the Cell (I2BC), CEA, CNRS, Univ. Paris-Sud, Université Paris-Saclay, 91198, Gif-sur-Yvette Cedex, France; 2 Institut Jean-Pierre Bourgin (IJPB), INRA - AgroParisTech, 78026 Versailles Cedex, France; 3 Light Microscopy Facility, Imagerie-Gif, Institute for Integrative Biology of the Cell (I2BC), CEA, CNRS, Univ. Paris-Sud, Université Paris-Saclay, 91198, Gif-sur-Yvette Cedex, France

**Keywords:** Microtubule, +TIPs (plus-end-tracking proteins), EB1 (end-binding protein 1), Microtubule bundling, Microtubule-network organization, Root growth

## Abstract

Microtubules are involved in plant development and adaptation to their environment, but the sustaining molecular mechanisms remain elusive. Microtubule-end-binding 1 (EB1) proteins participate in directional root growth in *Arabidopsis thaliana*. However, a connection to the underlying microtubule array has not been established yet. We show here that EB1 proteins contribute to the organization of cortical microtubules in growing epidermal plant cells, without significant modulation of microtubule dynamics. Using super-resolution stimulated emission depletion (STED) microscopy and an original quantification approach, we also demonstrate a significant reduction of apparent microtubule bundling in cytoplasmic-EB1-deficient plants, suggesting a function for EB1 in the interaction between adjacent microtubules. Furthermore, we observed root growth defects in EB1-deficient plants, which are not related to cell division impairment. Altogether, our results support a role for EB1 proteins in root development, in part by maintaining the organization of cortical microtubules.

This article has an associated First Person interview with the first author of the paper.

## Abstract (precis)

**Summary:** EB1 proteins affect cortical-microtubule bundling and organization in *Arabidopsis thaliana*, without significant modulation of microtubule dynamics. They also participate in root growth, further linking microtubules to plant development.

## INTRODUCTION

Tightly controlled cell expansion is crucial for plant development and adaptation to environmental conditions. An increasing amount of evidence suggests that the microtubule (MT) cytoskeleton plays a major role in this process (Andreeva et al., 2010; Anthony and Hussey, 1999; Bisgrove et al., 2008; Green, 1962; Hamada, 2014; Ishida et al., 2007; Sedbrook et al., 2004), however, the sustaining molecular mechanisms remain poorly described.

In most interphase plant cells, MTs are generally organized in a parallel manner at the cell cortex, beneath the plasma membrane (Green, 1962; Ledbetter and Porter, 1963). During cell expansion in roots and etiolated hypocotyls, cortical MTs are oriented transversely to the elongating axis (Granger and Cyr, 2001; Lloyd et al., 1985; Sugimoto et al., 2000). This particular organization allows them to contribute to the cell wall architecture by ensuring the guidance of cellulose synthase complexes as they synthesize cellulose microfibrils (Baskin, 2001; Crowell et al., 2009; Fujita et al., 2011; Gutierrez et al., 2009; Paredez et al., 2006). Hence, MTs are thought to sustain the assembly of the cell wall with anisotropic mechanical properties that favor cell elongation rather than widening.

Cortical MTs are assembled into bundles of various sizes (Ledbetter and Porter, 1963), which may be critical for the maintenance of the overall MT network architecture in plant cells (Smertenko et al., 2004). In spite of these early observations, the mechanisms of bundle formation are not yet well understood, but they could imply a reduction of the remarkable MT flexural rigidity (Gittes et al., 1993), mediated by associated factors (Ambrose et al., 2011; Portran et al., 2013).

Though the MT network looks tightly organized in plant cells, it remains highly dynamic; a remarkable behavior that allows MTs to change their 3D organization in response to environmental cues or cellular requirements (Lindeboom et al., 2013; Zhang et al., 2013). In both animals and yeasts, the end-binding-1 protein (EB1) accumulates at growing MT plus-ends where it regulates MT dynamics and polarization (Akhmanova and Steinmetz, 2008, 2010; Coquelle et al., 2009; Galjart, 2010; Guesdon et al., 2016; Morrison, 2007), thus contributing to the shaping of MT arrays in both interphase and mitosis (Lee et al., 2000; Rogers et al., 2002; Tirnauer et al., 2002; Wen et al., 2004). Three EB1 orthologs have been identified in *Arabidopsis thaliana* and other related plants (Bisgrove et al., 2008; Chan et al., 2003; Gardiner and Marc, 2003; Komaki et al., 2010; Mathur et al., 2003; Meagher and Fechheimer, 2003). AtEB1 proteins are very similar to each other and to animal EB1 counterparts, except divergent C-termini, suggesting specific plant properties (Dixit et al., 2006; Komaki et al., 2010). AtEB1a and AtEB1b are cytoplasmic and localize to the growing MT plus-ends (Abe and Hashimoto, 2005; Chan et al., 2003; Dhonukshe et al., 2005; Dixit et al., 2006; Mathur et al., 2003; Van Damme et al., 2004), whereas AtEB1c is confined into the nucleus and seems to play a role in mitotic-spindle assembly and positioning (Komaki et al., 2010). AtEB1b contributes slightly to MT dynamicity (Galva et al., 2014), but the cortical-MT network, in both root and etiolated hypocotyl cells, seems to be unaffected in *eb1* mutants (Bisgrove et al., 2008; Galva et al., 2014; Gleeson et al., 2012; Komaki et al., 2010). Nevertheless, root growth is altered in *eb1b* mutants (Bisgrove et al., 2008; Galva et al., 2014; Gleeson et al., 2012), which puts into question the involvement of MTs in cell expansion, organ growth and morphogenesis in plants as previously mentioned. This prompted us to thoroughly analyze the cortical-MT network in elongating cells of a cytoplasmic-EB1-deficient plant line (*eb1a-2 eb1b-3*) (Komaki et al., 2010) (see plant lines in Table S1).

We show here a moderate but significant disorganization of the cortical MT network in elongating cells of *eb1a-2 eb1b-3* double-mutant plants. This phenotype is accompanied by an apparent reduced MT bundling and a modified cell wall architecture, but it is not associated with a significant change in MT dynamicity. In addition, plants lacking functional cytoplasmic-EB1 proteins display a root length reduction, suggesting that AtEB1a and AtEB1b might contribute to root growth through the maintenance of the MT network organization.

## RESULTS AND DISCUSSION

### AtEB1a and AtEB1b contribute to the microtubule network architecture

**Fig. 1.**
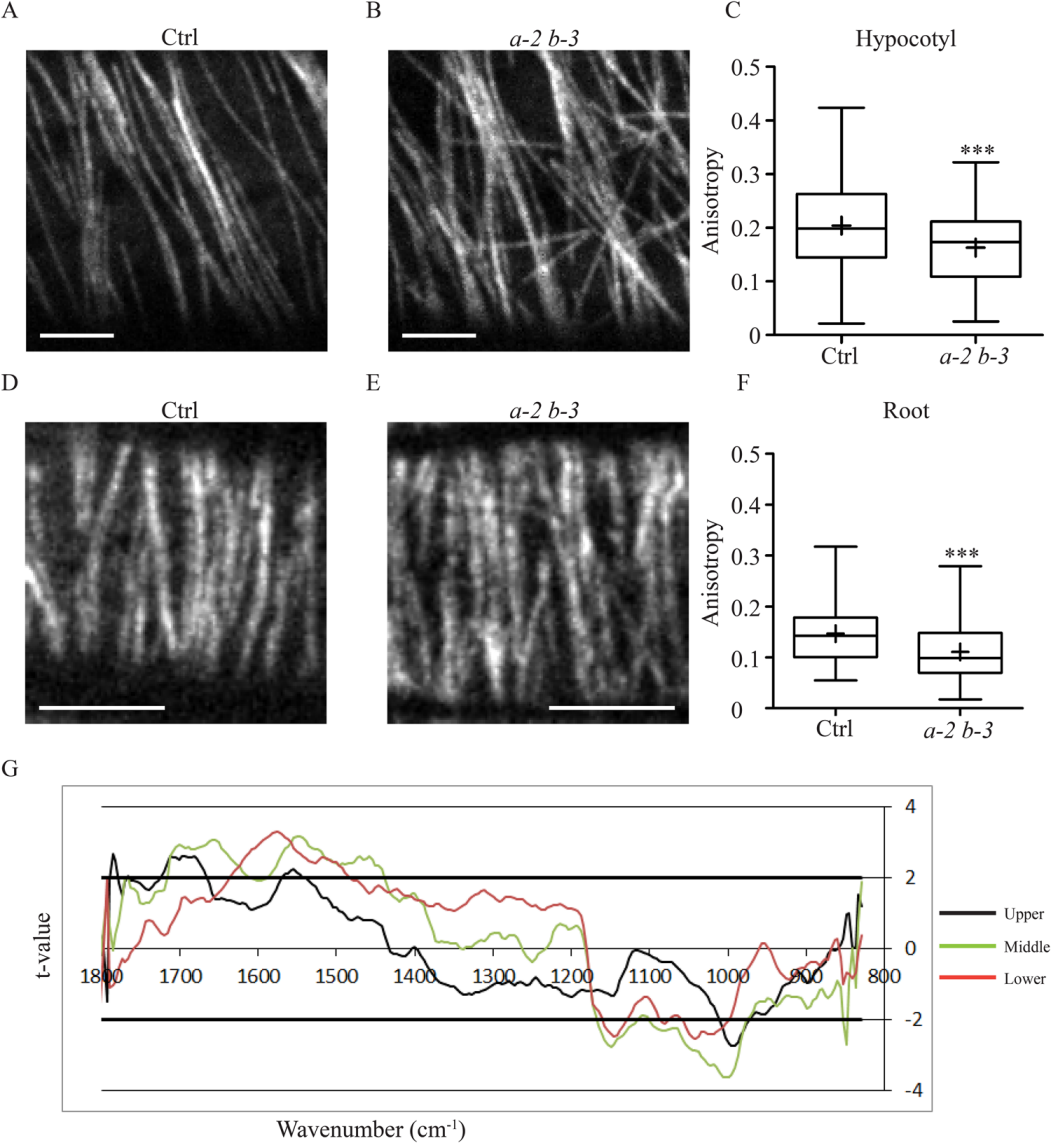
**Microtubule network disorganization in *eb1a-2 eb1b-3* double mutant.** Representative pictures of GFP-tubulin-labeled MTs in elongating epidermal cells from etiolated-hypocotyl (A,B) and root (D,E). The elongation axis is horizontal. Scale bar: 5 µm. In the double mutant (B,E) the MT network is disorganized compared to the control (A,D) as shown by the quantification of MT network anisotropy (C,F) in etiolated-hypocotyl cells (C) and in root cells (F) for both control (*n*=109 and *n*=132) and *eb1a-2 eb1b-3* (*n*=77 and *n*=112) genotypes. Crosses represent the mean, bars represent the median, and whiskers indicate minima and maxima. Asterisks indicate statistically significant differences (*P*=0.0009, *t*-test, in C and *P*<0.0001, *t*-test, in F) according to a Mann–Whitney U-test. (G) Student's *t*-test: *t-*value for the comparison between FT-IR spectra of control (Col0) and *eb1a-2 eb1b-3* plant lines plotted against the wavenumber (x-axis). FT-IR spectra were obtained from the upper part (black line), the middle part (green line) and the lower part (red line) of growing hypocotyl of plants that do not express GFP-fused tubulin. Horizontal thick lines indicate the significance limit values (*P*=0.95).

Growth defects have been reported for *eb1a/b* mutant *A. thaliana* plants, without a clear detailed analysis of the sustaining cortical MT network (Bisgrove et al., 2008; Gleeson et al., 2012). To investigate the role of AtEB1a and AtEB1b in the organization of cortical MTs, epidermal elongating cells expressing 35S::GFP::TuA6 (Komaki et al., 2010) were observed alive by spinning-disk confocal microscopy. In the upper etiolated hypocotyl and in the elongation zone of root tip, cells grow rapidly, usually displaying a typical transverse array of parallel MTs (Fig. 1A,D) (Granger and Cyr, 2001; Lloyd et al., 1985; Sugimoto et al., 2000). However, in *eb1a-2 eb1b-3* double-mutant plants, the MT network appears partially disorganized with less parallel fibers (Fig. 1B,E). To quantify this phenomenon, we compared the average anisotropy value of cortical MT arrays between the *eb1a-2 eb1b-3* double mutant and the control (both expressing GFP::TuA6), using the ImageJ Plug-in FibrilTool (Boudaoud et al., 2014)*.* Anisotropy measurement may vary from 0 to 1. A value of 0 indicates a totally disorganized (isotropic) array and a value of 1 indicates a perfectly ordered network with parallel fibers. One should mention that the latter case never happens with biological samples and, in our hands, the highest anisotropy value was 0.4, which is consistent with published values for MTs (Boudaoud et al., 2014). In control plants, we obtained an average anisotropy of 0.20±0.08 in hypocotyls and of 0.15±0.05 in roots (mean±s.d.) compared to 0.16±0.07 and 0.11±0.05 respectively in the double mutant (Fig. 1C and F respectively, see Fig. S3 for examples). The differences are statistically significant according to a Mann-Whitney test with α=0.01 and were confirmed by a frequency distribution analysis of cells according to their anisotropy value (Fig. S1). Nevertheless, single mutants *eb1a-2* and *eb1b-3* (expressing GFP::TuA6) do not display obvious alteration of their MT organization (Fig. S2A-D), suggesting a redundancy between both proteins. These results indicate that the cortical MT network is partially disorganized in growing cells of plants lacking functional cytoplasmic-AtEB1 proteins.

Cortical MTs guide the movement of cellulose synthase complexes within the plasma membrane, determining the quasicrystal organization of cellulose microfibrils in the cell wall (Baskin, 2001; Crowell et al., 2009; Fujita et al., 2011; Green, 1962; Gutierrez et al., 2009; Paredez et al., 2006). Thus, the disorganization of the cortical MT network in *eb1a-2 eb1b-3* double-mutant seedlings should come with an alteration of the cell wall architecture. To verify the cell wall integrity in *eb1a-2 eb1b-3* genotype, we performed Fourier-Transform InfraRed Spectroscopy (FT-IR) on hypocotyls. Spectra comparison between wild type Col-0 and double-mutant plants (both devoid of GFP::TuA6) reveals a significant difference at 990 cm^−1^, a characteristic feature of cellulose microfibrils 3D arrangement in the primary wall (Fig. 1G) (McCann et al., 1992; Mouille et al., 2003). A change in the cell-wall composition may explain such a difference. However, biochemical analysis of cell wall components does not reveal any significant difference between both genotypes, further supporting a disorganization of cell wall structure in EB1-deficient plants (Fig. S4). Thus, impairing cytoplasmic AtEB1 perturbs cell wall architecture, which is consistent with the cortical MT network disorganization in sustaining growing epidermal cells. Interestingly, the middle zone of hypocotyl displays the strongest cell wall disorganization (Fig. 1G), suggesting a slower cell wall maturation in double-mutant plants compared to wild type. The MT-array disorganization, induced by AtEB1a and AtEB1b mutations, might have slowed down the cell wall assembly process along the growing etiolated hypocotyl. However, this process is not abolished since the mature zone, at the bottom of hypocotyl, displays similar FT-IR spectra at 990 cm^−1^ between both genotypes (Fig. 1G). Alternatively, the highest disorganization in the middle zone may result from the accumulation over time of cell wall assembly defaults, which might be eventually suppressed at the bottom by a compensation mechanism. This phenomenon may partly account for the weakness of the phenotype, at the whole-plant level, of double or triple EB1 mutants (Bisgrove et al., 2008; Galva et al., 2014; Gleeson et al., 2012; Komaki et al., 2010). It is also worth noting that the cell wall of single *eb1a2* or *eb1b-3* mutant seedlings remains similar to Col-0 at 990 cm^−1^, further confirming a redundancy between both cytoplasmic-EB1 representatives in *A. thaliana* (Fig. S2E).

### AtEB1a and AtEB1b participate in microtubule bundle formation or maintenance

**Fig. 2.**
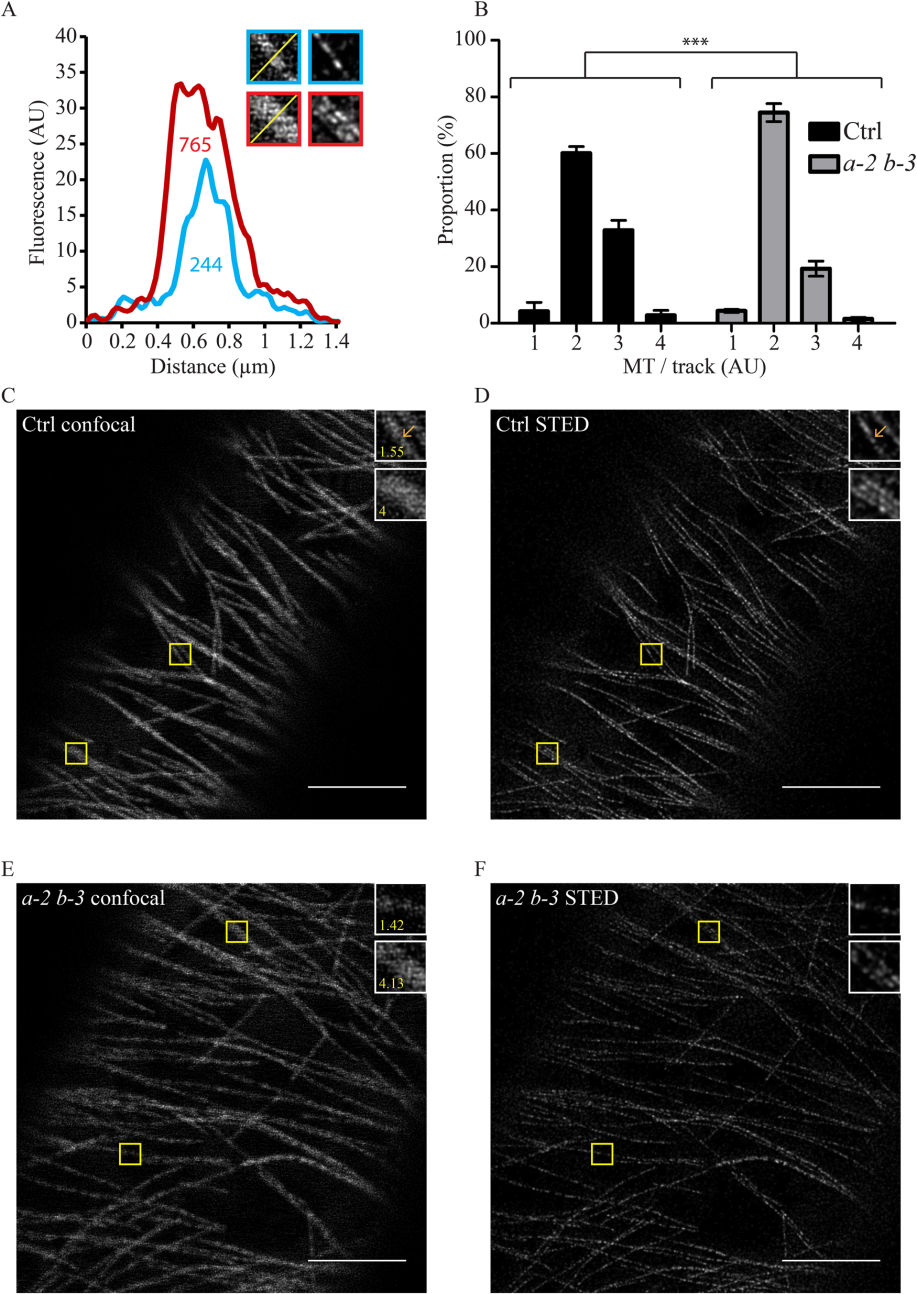
**Bundling is impaired in *eb1a-2 eb1b-3* double mutant.** (A) The graph shows that the number of MTs within a fluorescence track is proportional to the area under the plot profile [values indicated under the curves in arbitrary unit (AU)]. STED imaging reveals that the red-bordered track is made of at least three parallel MTs, whereas the blue-bordered one is made of at least one MT, which is consistent with the plot profiles and the related values. (B) Frequency distribution of the number of MTs per track in both genotypes (*n*=270 for the control and *n*=276 for the double mutant) based on the proportionality demonstrated in A. Asterisks indicate statistically significant differences (*P*=0.0006, Chi-square), bars represent s.e.m. Pictures showing confocal images (C,E) and STED images (D,F) of the same cell in control (C,D) and *eb1a-2 eb1b-3* double mutant (E,F) hypocotyl epidermal cells. Insets (yellow squares) show details of MT. Yellow number in insets indicate the number of MTs in the selection based on the method described in A. Scale bar: 5 µm.

To further explore MT arrays in the *eb1a-2 eb1b-3* double-mutant plants, we used stimulated emission depletion (STED) microscopy (Neupane et al., 2014) to achieve super resolution imaging on living seedlings. Previous studies showed that cortical MTs are assembled as bundles through tight lateral association (Ledbetter and Porter, 1963). This particular structure is frequent in nature and is believed to contribute to the overall MT array organization (Bratman and Chang, 2007; Dixit and Cyr, 2004b; Smertenko et al., 2004). Unfortunately, the resolution limit of classical light microcopy does not allow us to distinguish individual MTs from apparent bundles. Yet, STED microscopy, which improves the spatial resolution about five times (Neupane et al., 2014), allowed us to do so to a certain extent (Fig. 2). Careful analysis of bundles in both genotypes by comparing confocal (Fig. 2C,E) to STED acquisitions (Fig. 2D,F) revealed an apparent reduction of the average number of MTs per bundle in *eb1a-2 eb1b-3* double-mutant plants, compared to control. To quantify the bundling in both genotypes, we measured, on classical confocal images, the fluorescence profile across bundles as an indicator of the number of MTs. Indeed, as shown in Fig. 2A and Figs S5 and S6, the area under the fluorescence profile is proportional to the number of MTs in the track. If we make the hypothesis that the smallest area encountered in each condition corresponds to a single isolated MT (reference area), one can estimate the number of MTs per bundle by dividing the area under the fluorescent profile of each bundle by the reference area. The procedure was partly automatized with an ImageJ Macro that we designed (Fig. S7). Compared to control, we noticed a significant reduction of the percentage of tracks with more than two MTs in *eb1a-2 eb1b-3* double mutant (Fig. 2B). On average, one third of tracks have more than two MTs in control cells (35.64%). This proportion drops to one fifth in double-mutant cells (20.83%), without any change in the overall density of MTs (Fig. S8). These data suggest that bundling is affected in the absence of functional cytoplasmic-AtEB1 proteins. Yet, the link between network disorganization and bundling failure remains to be clarified.

Plant cells have two well-accepted mechanisms to generate MT bundles (Dixit and Cyr, 2004a,b; Ehrhardt, 2008; Fishel and Dixit, 2013): (1) a growing MT will zip along an existing older MT following their encounter or (2) during nucleation, the new MT will be created in a parallel manner to its mother MT, so lateral interaction all along the MT lattices is favored. On the one hand, because of its localization at the growing plus-end of MTs, EB1 could be involved in the zippering activity, either by itself or through the recruitment of partners. On the other hand, given that animal EB1 has already been shown to be involved in MT nucleation either *in vitro* (Vitre et al., 2008) or *in vivo* (Rogers et al., 2008), this function may be conserved in plant, as suggested before (Chan et al., 2003), and one can imagine that plant EB1 proteins favor the MT-nucleation from a mother MT at shallow-angle rather than steep-angle.

Altogether, these sub-cellular data show that cytoplasmic-AtEB1 proteins play a role in the formation and/or the maintenance of the MT network architecture.

### Microtubule dynamic instability is not significantly affected in *eb1a-2 eb1b-3* double-mutant plants

**Fig. 3.**
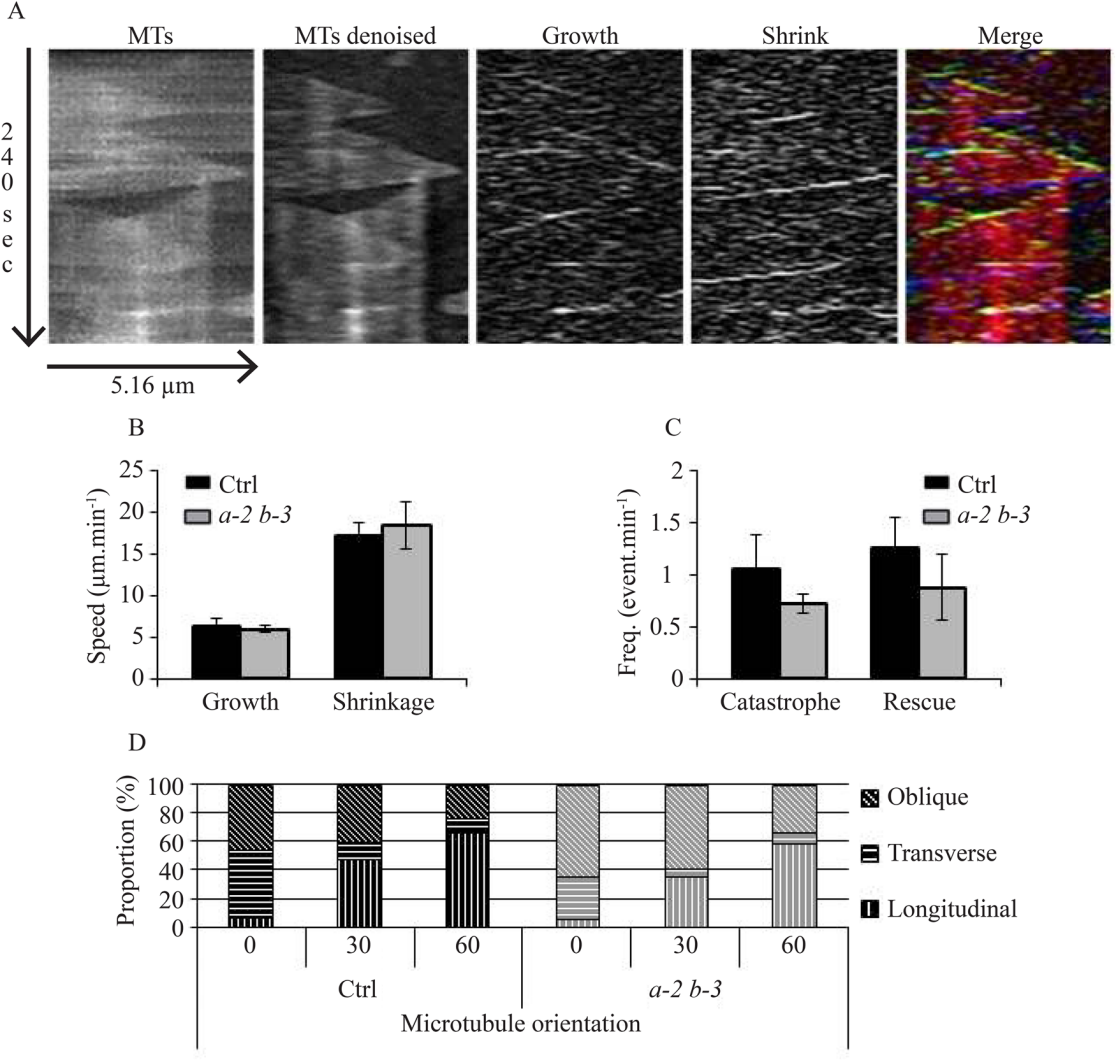
**The microtubule dynamic instability is not affected in *eb1a-2 eb1b-3* double mutant.** (A) Kymographs showing the same MT before and after image treatment (first and second panels). Image subtraction reveals growth and shrinking events (third and fourth panels). Color encoding facilitates the interpretation of kymograph. Growth events, shrinkage events and entire MTs appear in green, blue and red respectively on the merged image (fifth panel). (B) Growth and shrinking rates of MT plus-end in control and *eb1a-2 eb1b-3* double mutant. Bars indicate s.e.m. (C) Transition frequencies in control and *eb1a-2 eb1b-3* double mutant. Bars indicate s.e.m. Rates and frequencies came from 158 MTs in control and 125 MTs in *eb1a-2 eb1b-3*. Averaged measurements from eight plants have been compared using a Mann–Whitney test with α=0.05 and, according to this test, differences are not significant. (D) Percentage of cells with transverse (70°-90°), longitudinal (0°-20°) or oblique (20°-70°) MT network organization during auxin treatment (10 μM) in control and double-mutant plants (both expressing GFP-tubulin) after 0, 30, and 60 min of treatment. Differences are not significant according to Chi-squared *t*-test.

Fine-tuning of dynamic instability may account for MT array remodeling and subsequent cell polarization, morphogenesis and functions. Interestingly, the loss of function of the catastrophe factor ARK1 has been correlated to an increase of MT bundling in in *A. thaliana* (Eng and Wasteneys, 2014). EB1 proteins have been described in animals and yeasts as potent regulators of MT dynamics on its own and through other interacting partners (Akhmanova and Steinmetz, 2008, 2010; Coquelle et al., 2009; Vitre et al., 2008; Wieczorek et al., 2015). To investigate the role of AtEB1a and AtEB1b in the regulation of MT dynamics in *A. thaliana*, we carried out time-lapse acquisitions using total internal reflection fluorescence (TIRF) microscopy on hypocotyl epidermal cells, expressing GFP::TuA6, with *eb1a-2 eb1b-3* double-mutant and Col-0 backgrounds. After photobleaching an area of interest, making the analysis easier, we recorded 5 min movies, with one image every 2 s. After thorough picture treatments (see Fig. 3A and the Materials and Methods for details), we used KymoToolBox ImageJ Plug-In to determine the parameters of dynamic instability of MT plus-end. Growing and shrinking rates did not vary between both genotypes with average values that are consistent with previous studies (Fig. 3B; Table S2). However, catastrophe and rescue frequencies are slightly reduced in double-mutant plants compared to control, from 0.776 to 0.659 event.min^−1^ and from 1.152 to 0.928 event.min^−1^ respectively (Fig. 3C, detailed values in Table S2). Though consistent with a recent publication based on the hypomorphic *eb1b-2* mutant (Galva et al., 2014), these results are not significant according to a Mann-Whitney test, and thus are not likely to account for the MT network disorganization and for the cell wall architecture modification in EB1-deficient plants.

MT dynamicity is required for cortical-MT network re-orientation since, during this process, the new organization is appearing while the former one is disassembling (Lindeboom et al., 2013; Zhang et al., 2013). Along this line, we noticed that the reorientation of the MT network is triggered by high concentrations of auxin in *eb1a-2 eb1b-3* double-mutant plants, in a manner similar to control plants (Fig. 3D), confirming that MTs are still dynamic enough to respond to such an environmental change. Altogether, our data indicate that AtEB1a and AtEB1b do not play a crucial role in MT dynamic instability, unlike other eukaryotic counterparts (Coquelle et al., 2009).

### *eb1a-2 eb1b-3* double-mutant plants display skewed and shorter roots

*eb1b* hypomorphic mutant plants display roots that deviate toward the left on vertical and inclined plates (Bisgrove et al., 2008). This particular behavior has lately been linked to a possible increased sensitivity to mechanical stimulation (Gleeson et al., 2012). This moderate root skewing is the only phenotype previously described at whole-plant level as a consequence of *eb1-b* mutation, without clear connection to the underlying MT network (Bisgrove et al., 2008; Galva et al., 2014; Gleeson et al., 2012).

**Fig. 4.**
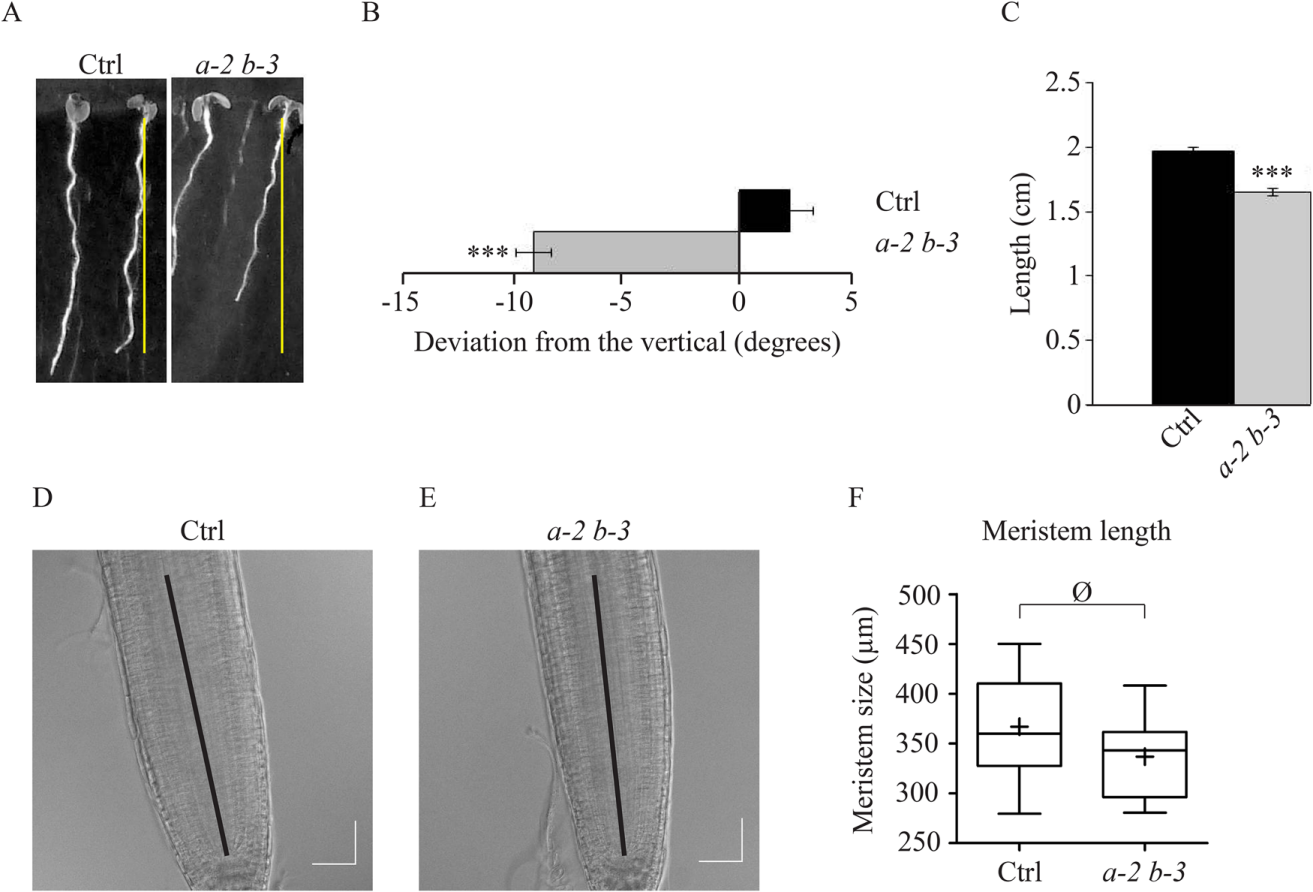
***eb1a-2 eb1b-3* double-mutant plants display skewed and shorter roots.** (A) Typical pictures illustrating root skewing and root length for control and *eb1a-2 eb1b-3* double-mutant plants (both expressing 35S::GFP:TuA6). (B) Measurement of the root-skewing angle for both genotypes, *n*=89 for the control and *n*=84 for the double mutant. (C) Measurement of the root length for both genotypes, *n*=93 for the control and *n*=76 for the double mutant. Asterisks represent statistically significant differences (Kruskal–Wallis, α=0.01). Bars indicate s.e.m. (D) Root apical meristem from Col-0 and (E) root apical meristem from *eb1a-2 eb1b-3* double-mutant plants after 7 days of growth on 0.3% agar medium. (F) Box plot showing the root apical meristem length in Col-0 and double mutant, measured from the quiescent center to the beginning of the elongation zone. Crosses represent the mean, bars represent the median and whiskers indicate minima and maxima. *n*=17 for Col-0 and *n*=14 for the double mutant. Difference is not significant according to a *t*-test with α=0.05. Scale bar: 50 μm.

To investigate the consequence of the *eb1a-2 eb1b-3* double loss of function mutation at the whole-plant level, we grew double-mutant plants on vertically oriented plates containing agar-solidified nutrient medium. Double-mutant plants, expressing GFP-labeled tubulin, exhibit deviated root toward the left by an average angle of −7.3±5.9° (mean±s.d.), unlike control plant roots, expressing the same GFP-labeled tubulin, that are oriented to the right with an average angle of 1.8±8° (Fig. 4A,B). Given that this skewing phenotype is worsened with the medium hardness for *eb1a-2 eb1b-3* plants, compared to control ones (Fig. S9A,C), our results suggest that *eb1a-2 eb1b-3* plants are hypersensitive to mechanical cues as proposed for mutants with reduced expression of AtEB1b (Gleeson et al., 2012). Though impaired MT stability has already been related to a root skewing phenotype (Galva et al., 2014; Nakajima et al., 2006; Sedbrook et al., 2004), a direct contribution of the MT network architecture cannot be excluded. Given that bundling increases MT-flexural rigidity (Portran et al., 2013), a weakened mechanical resistance of the MT network, due to a reduced bundling activity, in root tip cells, could account for such a skewing phenotype. However, this cannot be confirmed since *eb1b-3* single-mutant plants display a significant root skewing without any visible MT disorganization (Fig. S2C-F)*.*

Interestingly, we noticed a significant reduction of root length in *eb1a-2 eb1b-3* mutants, compared to the control, suggesting a cell growth inhibition in EB1-deficient seedlings (Fig. 4C). Supporting this hypothesis, root apical meristems in *eb1a-2 eb1b-3* mutant plants are similar in size to those of control plants indicating that the phenotype is due to a cell elongation defect rather than a mitotic disorder (Fig. 4D-F). This root-size reduction does not depend on medium hardness and, whilst favoring root skewing on its own, *eb1b-3* mutation is not sufficient to reduce root length (Fig. S2G). These results indicate that *eb1a* and *eb1b* are not completely redundant and that both latter phenotypes, root skewing and root-size reduction, are not necessarily linked to each other. In addition, it is worth noting that both skewing and shortening phenotypes are independent of the expression of 35S::GFP::TuA6, since *eb1a-2 eb1b-3* mutant plants in Col-0 background display comparable features (Fig. S2F)*.* This is the first time that a reduction in root length is observed in a double mutant of EB1a and b. This phenotype was observed in two different growth chambers, by various examiners, so we believe that the discrepancy with previous studies (Bisgrove et al., 2008) might be due to the genetic background (Columbia versus Wassilewskija) or to the alleles used (tDNA insertion in introns versus exons versus premature stop codon).

In conclusion, we have provided, for the first time, evidence for a role of cytoplasmic-EB1 proteins in MT organization and bundling in growing plant cells without modulating MT dynamics in a significant manner. Furthermore, whilst MT network disorganization cannot account on its own for root skewing in cytoplasmic-EB1-deficient plants, our results suggest a possible relationship between the architecture of the cortical-MT array and root growth, mediated by AtEB1a and AtEB1b. The relatively mild phenotypes described here do not preclude an essential role for EB1 proteins in plants. They may be crucial in local MT-array adjustments in response to environmental constraints that we did not reproduce in our experiments. Of course, we cannot exclude the possibility that EB1 acts through an MT-independent process to account for the root growth phenotype we observed in *eb1a-2 eb1b-3* plants. In any case, these mild phenotypes allow to dwell on more mechanistic details than more severe ones leading to lethality or to dramatic pleiotropic impairments. Hence, tuning experimental conditions will be critical to shed more light on EB1 functions in plant survival and adaptation to external stresses.

## MATERIALS AND METHODS

### Arabidopsis lines

GFP labeled lines (35S::GFP:TuA6 and 35S::GFP:TuA6 *eb1a-2 eb1b-3*) and single *eb1a-2* and *eb1b-3* mutants were gifts from Prof. T. Hashimoto (Komaki et al., 2010; Ueda et al., 1999). An *eb1a-2 eb1b-3* double mutant (without GFP:TuA6) was generated by crossing the corresponding single-mutant lines (Table S1). A single *eb1b-3* mutant line stably expressing 35S::GFP:TuA6 was generated by crossing the 35S::GFP:TuA6 line (Ueda et al., 1999) with the single *eb1b-3* mutant line (Komaki et al., 2010).

We performed PCR using the following conditions: 30 cycles; 30 s at 92°C; 30 s at 55°C and 72°C for 30 s to identified homozygous seedlings. The first cycle was preceded by 5 min at 92°C and the last cycle was followed by 5 min at 72°C.

*eb1a-2* T-DNA insertion was checked using 5′-AAC GTC CGC AAT GTG TTA TTA AGT TGT C -3′ and 5′-GCA AAC AAA CAA TAA CCA TC -3′PCR primers. MseI enzyme was used (Thermo Scientific; FD2174), on PCR amplicons produced with 5′- TTG AAG CAA AGA ACG AGT ACG A -3′ and 5′- TGG AAG TAG CCA CAG GAG GA -3′ primers, to confirm the presence of the MseI restriction site created in the EB1b gene by *eb1b-3* point mutation.

### Growth conditions

Seeds were surface sterilized in 70% (v/v) ethanol and 0.05% (v/v) Tween-20 for 10 min and stratified on growth medium for 12 h at 4°C in the dark. Growth medium: half strength Murashige and Skoog solid medium composed of 2.15 g.l^−1^ MS Basal Salt Mixture (Sigma-Aldrich; M5524), 5 g.l^−1^ Sucrose (Merck, Darmstadt, Allemagne; 1.07651.1000), 0.59 g.l^−1^ 2-(N-morpholino)ethanesulphonic acid, pH 5.6 and 3 g.l^−1^, 6 g.l^−1^ or 9 g.l^−1^ of Gelzan (Sigma-Aldrich, G1910) or PhytoAgar (Duchefa Biochemie, Haarlem, The Netherlands; P1003.1000). Seedlings were grown vertically for 4 days in the dark at 20°C after exposure to light for 6 h (hypocotyl investigations) or 7 days at 20°C with a 16-h light/8-h dark photoperiod (root investigations) in growth chamber (Panasonic; MLR-352). Plates were sealed with gas-permeable medical tape (Urgo Medical, Paris, France).

### Whole-plant investigation

After 7 days of growth, plates were opened, turned to horizontal position and photographed, with seedlings facing the camera.

### Light microscopy

All observations were done on epidermal cells of elongating zone of hypocotyl or root.

For MT network organization, images were acquired using a Nipkow Spinning Disk confocal system (Yokogawa, Musashino, Tokyo, Japan; CSU-X1-A1) mounted on a Nikon Eclipse Ti E inverted microscope, equipped with Nikon APO TIRF X100/1.49 oil immersion objective. We used a single band 491 nm (Semrock, New York, USA) dichroic mirror combined with a Bandpass 525/45 nm (Semrock) emission filter.

For the measurement of dynamic-instability parameters, movies were acquired by TIRF using Nikon Eclipse Ti microscope equipped with a Nikon TIRF Illuminator Unit and with an APO TIRF X100/1.49 oil immersion objective. We used a Triple band 405/491/561 nm (Semrock) dichroic mirror and a Bandpass 525/50 nm (Chroma, Bellows Falls, USA) emission filter. Photobleaching parameters were controlled by iLAS2 module (Roper Scientifics, Trenton, USA). The whole system was driven by MetaMorph software (Molecular Devices, San Jose, USA). Images were recorded with a CoolSNAP HQ2 CCD camera (Photometrics, Tucson, USA). Blue (491 nm Cobolt Calypso DPSS, 100 mW; Cobolt, Solna, Sweden) laser was used for excitation of the GFP and photobleaching.

For super resolution STED microscopy on living plants, we used a DMi 6000 TCS SP8X – gated STED (LEICA, Wetzlar, Allemagne) microscope equipped with a HCX PL APO 100×/1.40 STED oil immersion objective and a continuous 592 nm depletion laser. To obtain STED and confocal acquisitions of the same field of view, we used the sequential mode. During STED first sequence, a 592 nm depletion laser was combined with a 491 nm excitation wavelength. During confocal acquisitions, depletion laser was turned off. Both images were obtained with four times line accumulation and the emission was collected on GaAsP Hybrid (Hamamatsu Photonics K.K., Hamamatsu City, Japan) detector in time-gating mode (between 1 ns and 6 ns). Numerical zoom (×5.7) and image size (1024×1024) were chosen to obtain a final pixel size of 20 nm. Scanning speed was 400 Hz to deal with living sample movement and signal strength. Confocal and STED images were denoised using Huygens Essential Package (Scientific Volume Imaging, Hilversum, The Netherlands).

In every case, to avoid stress-related MT re-organization, the observation time never exceeded 20 min after the seedlings were mounted in the dark between slides and coverslips in liquid half M&S medium. For auxin treatment, 4-day-old etiolated seedlings were mounted in the dark under green light, between slides and coverslips, in half M&S medium containing 10 µM of Indole-3-acetic acid (IAA), and then slides were kept vertical in the dark at room temperature for 0 to 60 min and observed for less than 20 min.

### FT-IR

4-day-old etiolated plants were used for FT-IR acquisitions. Seedlings were fixed in 70% v/v ethanol solution overnight and transferred in water overnight the day before data acquisition. The rehydrated seedlings were then laid down on gold-coated microscope slides and left to dry for 20 min at 37°C. The zone targeted for the acquisition of spectra was the top (below the hook), middle and bottom of the hypocotyl. Spectra were collected using a Nicolet iN10 MX FT-IR Microscope (Thermo Scientific). Eight interferograms were collected in the reflection mode with 8 cm^−1^ resolution and co-added to improve the signal-to-noise ratio of the spectrum.

### Biochemical analysis of cell-wall components

The analyses of polysaccharides were performed on an alcohol insoluble residue (AIR) and prepared as follows. About hundred mg (FW) of roots were washed twice in 4 volumes of absolute ethanol at 100°C for 15 min, then rinsed twice in 4 volumes of acetone at room temperature for 10 min and left to dry under a fume hood overnight at room temperature. Samples were then treated with 0.1 M NaOH at 4°C overnight. After centrifugation for 10 min at max speed the supernatant was discarded and the pellet was washed twice with 2.5 M trifluoroacetic acid at room temperature in order to perform the neutral monosaccharide composition (see below). Pectins were extracted with 0.5% ammonium oxalate at room temperature for 2 h according to (Neumetzler et al., 2012). Uronic acid content was measured from saponified, ammonium oxalate-extracted pectins following the method described in (Blumenkrantz and Asboe-Hansen, 1973). Neutral monosaccharide composition was performed on 5 mg of the extracted cell wall fraction after hydrolysis in 2.5 M trifluoroacetic acid for 1.5 h at 100°C as described in Harholt et al. (2006). To determine the cellulose content, the residual pellet obtained after the monosaccharide analysis was rinsed twice with 10 volumes of water and hydrolyzed with H_2_SO_4_ as described in Updegraff (1969). The released glucose was diluted 500 times and then quantified using an HPAEC-PAD chromatography as described in Harholt et al. (2006).

### Image analysis

#### MT network disorganization

Individual cells were cropped from spinning disk images. We used FibrilTool ImageJ plug-in (Boudaoud et al., 2014) to quantify the organization of the MT array in each cell. The plug-in output is a value of anisotropy which may vary from 0 (isotropic or totally disorganized array) to 1 (perfect parallel MTs).

#### MT bundling

Classical scanning confocal images acquired immediately after STED images have been used to quantify the bundling of MTs in each genetic context (living seedlings). STED images were used as control to verify the accuracy of the quantification. Firstly, images from each genotype were stacked and normalized using the appropriate ImageJ functions [process→enhance contrast→normalize histogram (all stack +0.3% saturation)]. Quantification of bundling was then made using an ImageJ macro that we developed. The procedure is semi-automated, needing user intervention at some points. For each image, standard deviation to the mean of pixel intensity was subtracted, in order to get rid of the detector noise. Briefly, 50×100 pixels’ regions of interest (ROI) were centered manually on an MT fluorescent track. Areas under each MT portion were then measured using plot profile from the Gel Analyser ImageJ function. The macro output is a list of quantified areas, which were then normalized and analyzed using Excel (Microsoft) and Prism software (GraphPad Software).

An average of 10 portions of MTs have been analyzed in each image. Totals of 270 and 276 measurements have been performed for the control and for the double-mutant genotype respectively. In each data collection, results have been normalized by dividing areas by the smallest area. Hence, this ratio is a relative quantification of the number of MTs in each track analyzed. STED images allowed us to confirm that the number of MTs visible in STED correspond approximately to the ratio value (see Fig. 2A; Figs S5 and S6).

MT density was measured using STED images. Images were normalized using the normalize histogram function from ImageJ and then for each image the standard deviation to the mean was subtracted. MT average orientation was measured using the FibrilTool ImageJ plug-in (Boudaoud et al., 2014). Images were rotated accordingly to make the average orientation vertical. A horizontal intensity profile (width=20 pixels) was traced in the middle of each image. Values from the fluorescence profile were imported into Excel. The first derivative of the fluorescent profile was used to identify MTs. The number of MTs per cell was then divided by the length of the portion of the plot profile within the cell.

#### MT dynamics

We first de-noised movie pictures by subtracting standard deviation to the mean of pixel intensity from each image and by applying a difference of Gaussian filter (DOG). MTs growth and shrinking rates were then determined using KymoToolBox ImageJ plug-in (F. Cordelières). To make easier kymographs analysis, we performed image treatment, inspired from Lindeboom et al. (2013), in order to distinguish with false colors growth and shrinkage events. Briefly, consecutive image subtraction (I_n+1_ – I_n_) was used to reveal growing and shrinking MTs+ends as spots of positive or negative intensity. False colors were used to highlight MT body, growing-ends and shrinking-ends in red, green and blue respectively, in order to better distinguish stages transitions (catastrophe and rescue) on kymographs (Fig. 3A). See Table S2 for experimental values.

#### Auxin re-orientation assay

Each cell image was rotated to be oriented horizontally. Each cell was analyzed using FibrilTool ImageJ plug-in (Boudaoud et al., 2014). The MT network orientation angle was used to determine the organization of the MT network; transverse (70°-90°), longitudinal (0°-20°) or oblique (20°-70°).

#### Root skewing

We drew a line going through the collar and the root tip on each image of 7-day-old plants grown on vertical plates and measured the deviation angle from a vertical line using ImageJ build-in functions.

#### Root length

Root length was measured from images of 7-day-old plants grown on vertical plates. Pictures were analyzed with NeuronJ plugin (Meijering et al., 2004). Images were calibrated according to the ruler placed next to each plate on pictures.

#### Meristem size measurement

For root apical meristem size, images were acquired using SP8-X microscope (Leica) equipped with a Leica X40/1.30 oil immersion objective. We used 660 and 670 nm laser light with a PMT equipped for differential interference contrast (DIC) imaging. Seedlings were mounted in half M&S medium. Double-sided tape (500 µm thick) was used to ensure that the roots were not smashed during the mounting. Meristem size was estimated by measuring the distance between the cells in the quiescent center and the first elongated cell of the epidermis as represented by the thick black line in Fig. 4D,E.

#### Statistical analysis

Statistical Analyses were performed using R software for FT-IR data and Prism software (GraphPad Software) for all the other experiments.

#### MT network organization

Images analyzed came from two biological repetitions for hypocotyl and from three biological repetitions for the root. A minimum of 10 plants were analyzed for each genotype and each organ. 109 cells from the control and 77 cells from the double mutant were analyzed for hypocotyl and 132 cells from the control and 112 cells from the double mutant were analyzed for the root. Data normality was tested using the KS normality test, d'Agostino and Pearson omnibus normality test and Shapiro-Wilcoxon normality test. In the case of the hypocotyl, all three tests concluded that data fit a normal distribution, while all tests concluded that data did not fit a normal distribution for the root. Hence, we decided to use a non-parametric Mann–Whitney U-test with α=0.01 for both data collections. Differences between control and double mutant were significant in both organs with *P*=0.0009 for the hypocotyl and with *P*<0.0001 for the root.

#### Cell wall architecture

For each genotype, a total of 20 FT-IR spectra were acquired from five individual hypocotyls of four independent biological repeats. The collected spectra were baseline corrected and normalized as described before by Mouille et al., (2003). Wave numbers for which have absorbance values that are significantly different between genotypes, identified using a Student’s *t*-test.

#### MT bundle quantification

Data came from three biological repetitions. We analyzed 29 different cells from 10 plants, for each genotype for a total of 270 and 276 MTs analyzed for control and double mutant respectively. To facilitate data comparison, histogram of frequencies distribution upon the whole population with four classes; 1<…<2, 2<…<3, 3<…<4 and >4, has been used to compare the two distributions using a Chi-squared test and it appears that samples are significantly different with *P*=0.0006.

#### MT dynamic instability

Parameters of MT dynamic instability were determined from three biological repetitions, for a total of eight plants per genotype. One cell was analyzed for each plant. Twenty-nine MT tracks were analyzed in both genotypes. For each parameter (growth rate, shrinking rate, rescue frequency and catastrophe frequency) an averaged value was calculated for each plant and then the eight values obtained were compared using a non-parametric Mann–Whitney test with α=0.05. Differences between control and double mutant were not significant.

#### Auxin re-orientation assay

For each time point, at least 59 cells from 10 seedlings from two biological repetitions were analyzed. Frequencies at each time point were compared using Chi-squared *t*-test with α=0.05 and differences were not significant.

#### Root length and skewing

Data came from three biological repetitions. 76<*n*<104 for length; 84<*n*<103 for skewing. Normality of the data was tested using the KS normality test, d'Agostino and Pearson omnibus normality test and Shapiro-Wilcoxon normality test. The normality was not ensured for certain experiments, so that we decided to use a Kruskal–Wallis test with α=0.01. Significant differences are highlighted by a letter.

#### Meristem size measurement

Data come from 17 and 14 plants of Col-0 and *eb1a-2 eb1b-3* double-mutant genotypes respectively, from three biological repetitions. Data normality was tested using the KS normality test, d'Agostino and Pearson omnibus normality test and Shapiro-Wilcoxon normality test. All three tests conclude that data fit a normal distribution. Data were compared using a *t*-test with α=0.05.

## Supplementary Material

Supplementary information

First Person interview
