# Supplementary material for: EB1 contributes to microtubule bundling and organization, along with root growth, in Arabidopsis thaliana
Source: Biol Open. 2018 Jun 26;7(8):bio030510. doi: 10.1242/bio.030510 (PMC6124560; doi:10.1242/bio.030510)
Supplement: Supplementary information [file biolopen-7-030510-s1.pdf]

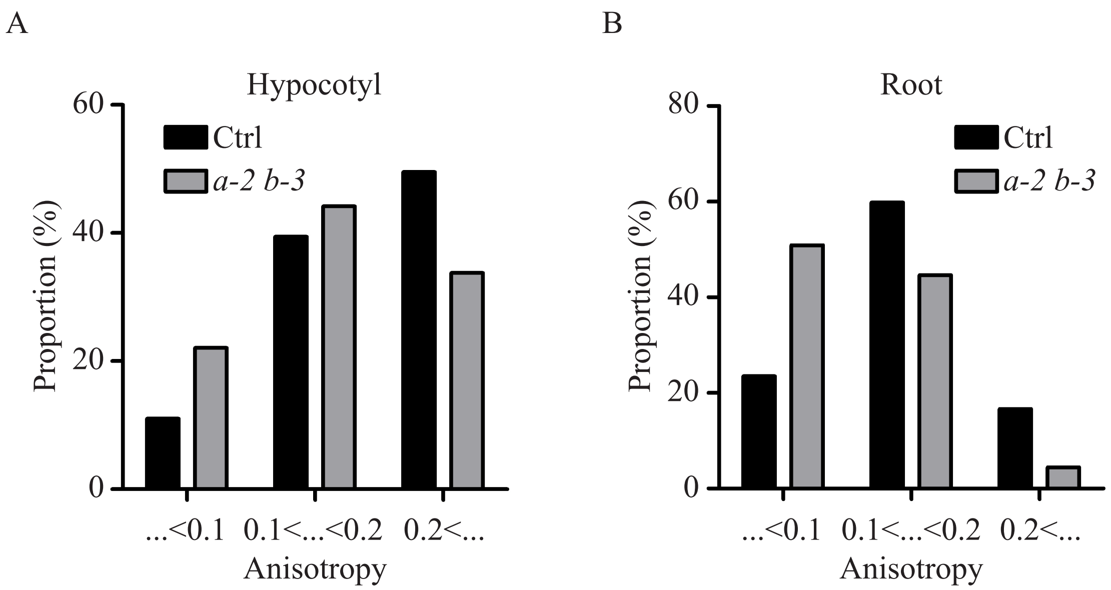

Figure S1: Frequency distribution of anisotropy

Frequency distribution of the anisotropy value in control and double-mutant cells (growing hypocotyl (A) and root cells (B)).The population is clearly enriched with small values of anisotropy in the double mutant. MT network anisotropy in etiolated-hypocotyl cells (A) and in root cells (B) for both control (n=109 and n=132) and *eb1a-2 eb1b-3* (n=77 and n=112) genotypes.

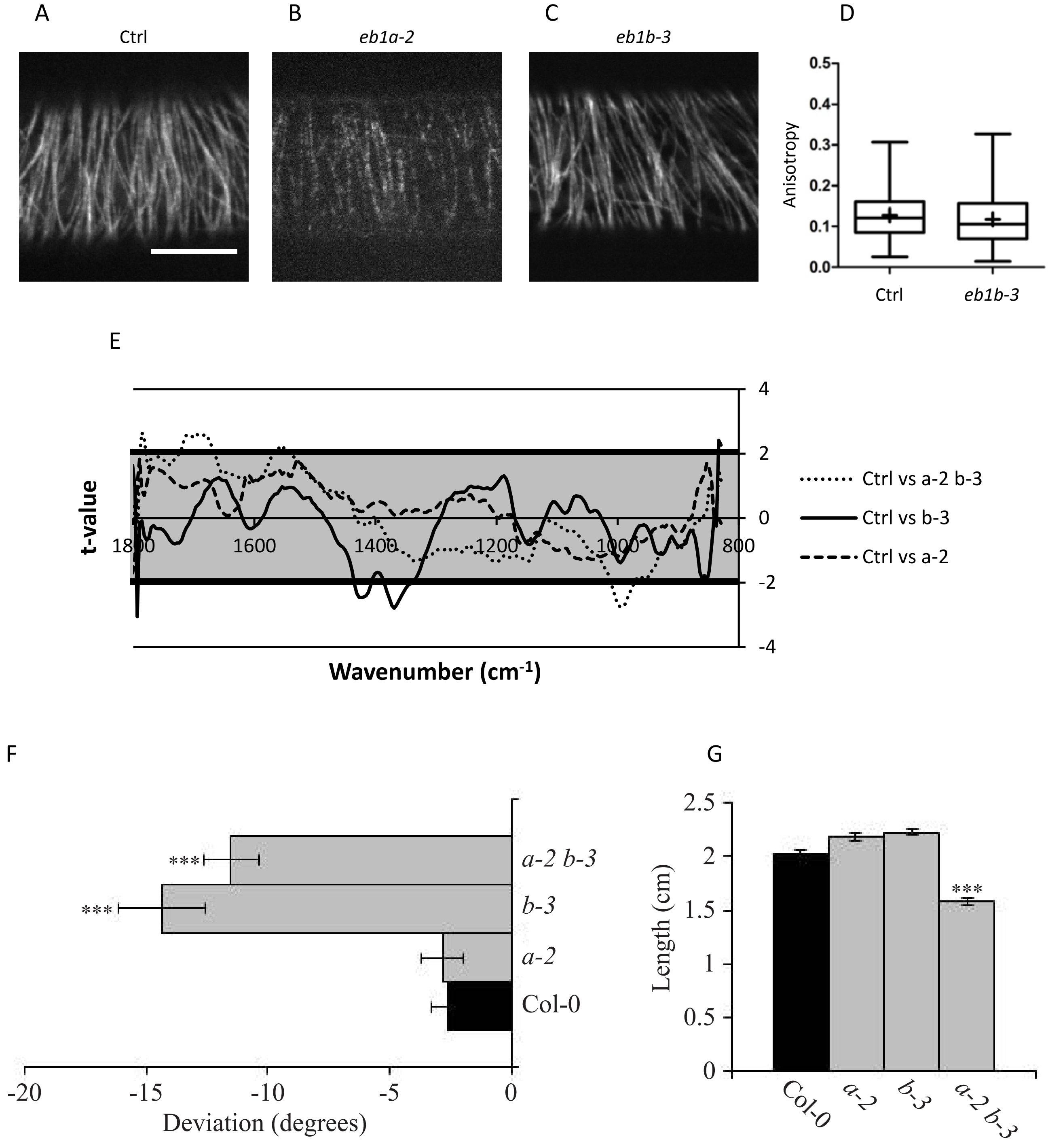

Figure S2

Biology Open • Supplementary information

Figure S2: cellular and macroscopic phenotypes of *eb1a-2* and *eb1b-3* single mutants

(**A, B and C**) Representative images of GFP-labeled microtrubules in elongating epidermal cells from etiolated-hypocotyl. The elongation axis is horizontal. Scale bar, 10µm. The GFP signal is of poor quality in the *eb1a-2* mutant line, impairing the image analysis process. The microtubule network seems correctly organized in both single mutant lines. This observation is supported by the MT-network anisotropy quantification shown in (**D**). Crosses represent the mean, bars represent the median, and whiskers indicate minima and maxima. n= 76 and 104 cells for control and *eb1b-3* respectively. Data come from 3 replicated experiments and from 9 and 7 plants respectively. (**E**) Student's t test: *t*-value for the comparison between control (Col0) and *eb1a-2* (square dotted line), *eb1b-3* (continuous line) and *eb1a-2 eb1b-3* (round dotted line) FT-IR spectra plotted against the wavenumber (x-axis). FT-IR spectra obtained in the upper part of the hypocotyl of plants that do not express GFP-fused tubulin. n=20 for each genotype. Horizontal thick lines (grey area) indicate the significance limit values (p=0.95). Root skewing (**F**) and root length (**G**) in plants lines that do not express GFP-fused tubulin. (**F**) Root skewing for single *eb1a-2* (n=83) or *eb1b-3* (n=60) mutants and for *eb1a-2 eb1b-3* (n=46) double mutant without the expression of the GFP-tubulin, compared with Col-0 (n=237). 7-day-old plants grown on 3g.L^{-1} agar medium. Asterisks indicate statistically significant differences according to a Mann-Whitney t-test with α=0.01. (**G**) Measurement of the root length for single *eb1a-2* (n=83) or *eb1b-3* (n=60) mutants and for *eb1a-2 eb1b-3* (n=43) double mutant without the expression of the GFP-tubulin compared with Col-0 (n=236). 7-day-old plants grown on 3g.L^{-1} agar medium. Asterisks indicate statistically significant differences according to a Mann-Whitney t-test with α=0.01. Error bars indicate SEM.

Biology Open • Supplementary information

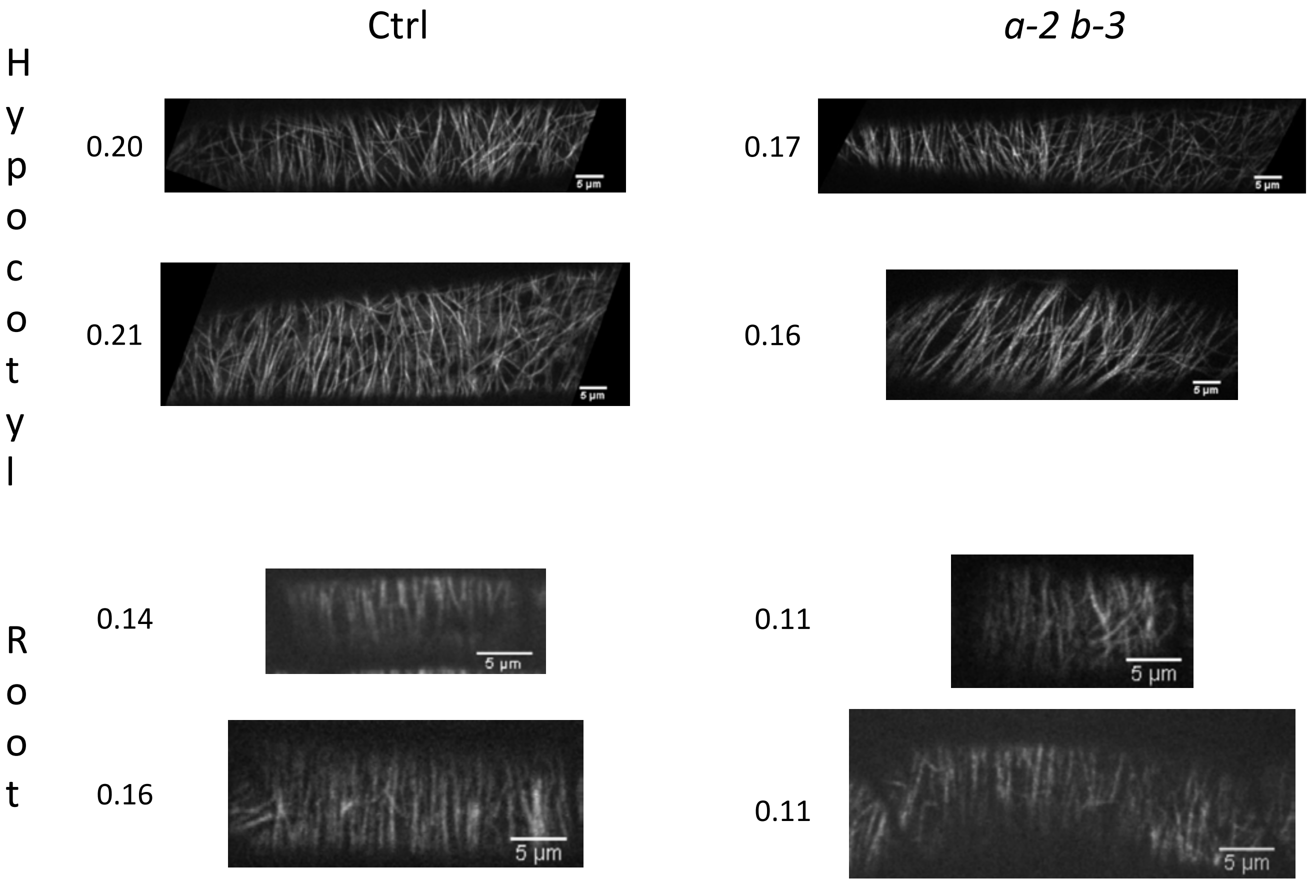

Figure S3: Representative images from the control and the double mutant plant showing microtubules network organization and the corresponding anisotropy value.

Biology Open • Supplementary information

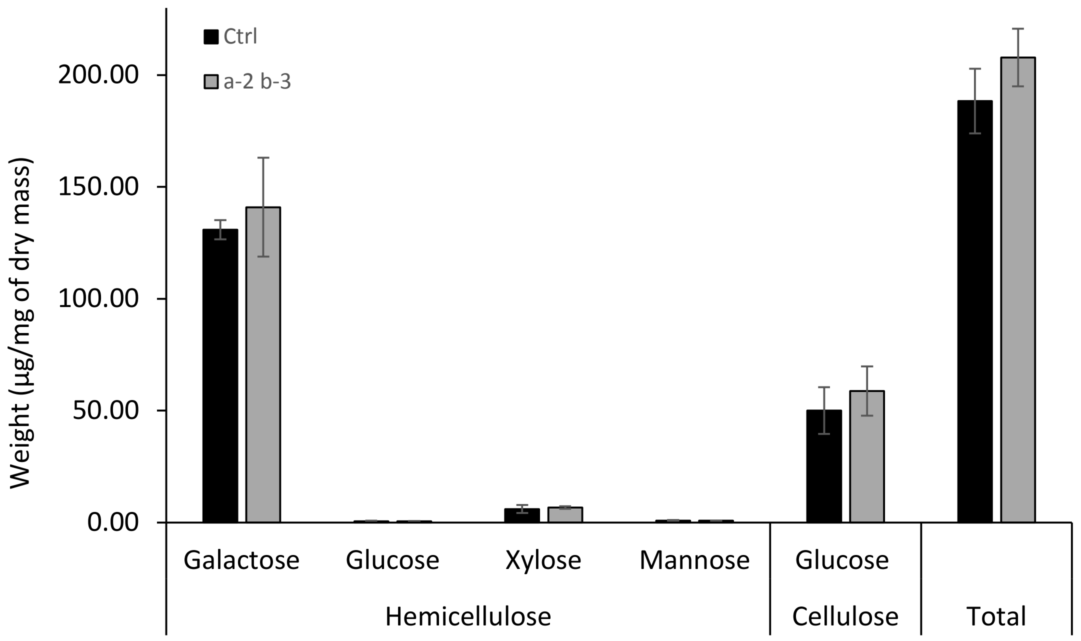

Figure S4: Monosaccharide compositional analysis of the cell wall residue of the root.

Neutral monosaccharides content were determined by HPLC (HPAEC-PAD). Uronic acid content was determined using a colorimetric method (MHDP). The cellulose content was determined using a modified Updegraff method (see Materials and Methods). Data are means of 4 independent biological repeats, in micro-gram per milligram of desiccated weight. Error bars = SD.

Biology Open • Supplementary information

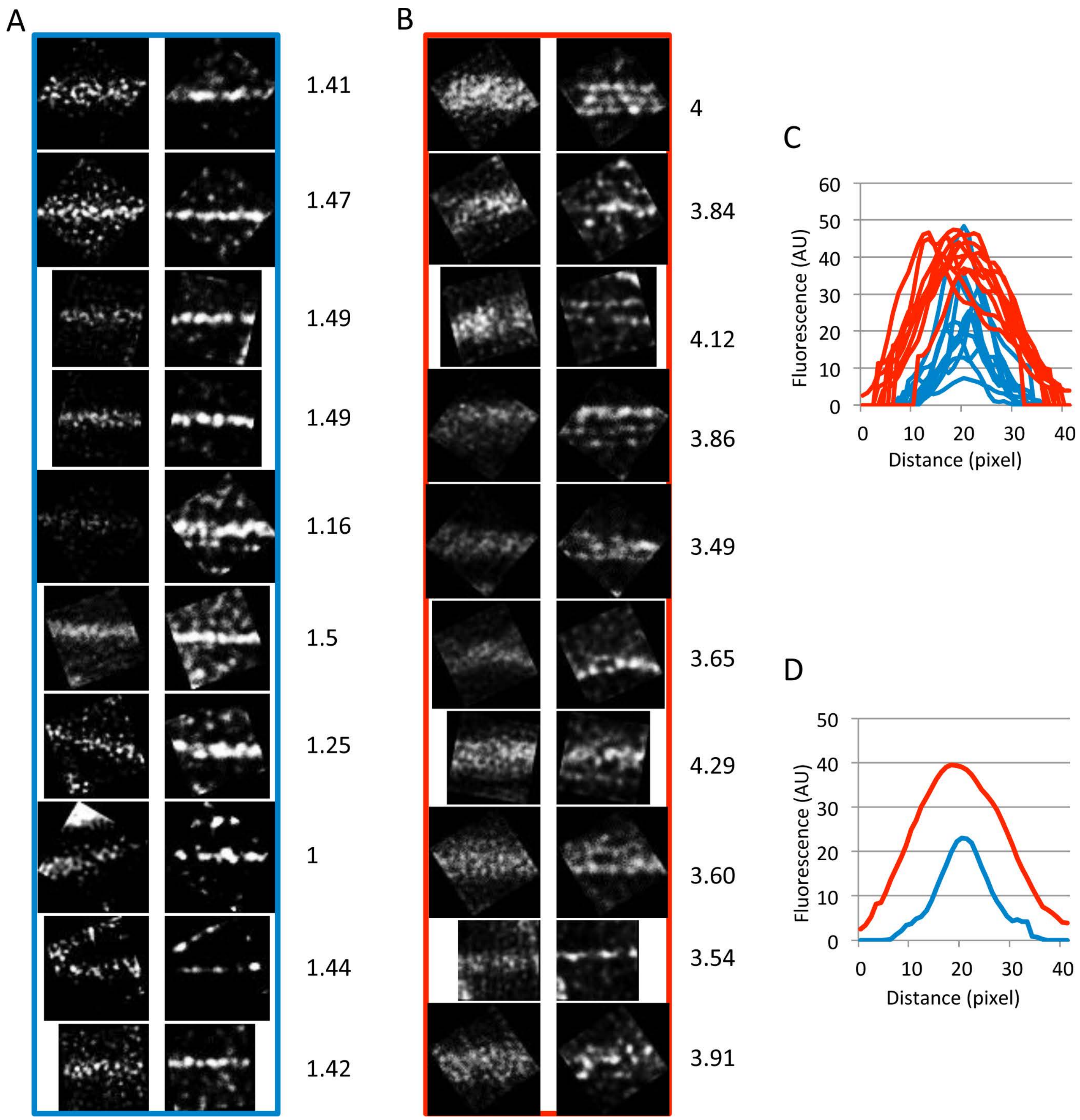

Figure S5: correlation between the integral of fluorescent signal and the number of microtubules in the track

The ten microtubules with the smallest area under the plot profile are shown in the blue box, the ten microtubules with the biggest area under the plot profile are shown in the red box. In each boxes, confocal (left column) and corresponding STED (right column) images are shown for each microtubule. Plot profile for each microtubule shown in (A) and (B) are presented in (C). Corresponding profiles are averaged in (D). Labeling next to (A) and (B) indicates the estimation of the number of microtubules in each track based on the area under the plot profile.

Biology Open • Supplementary information

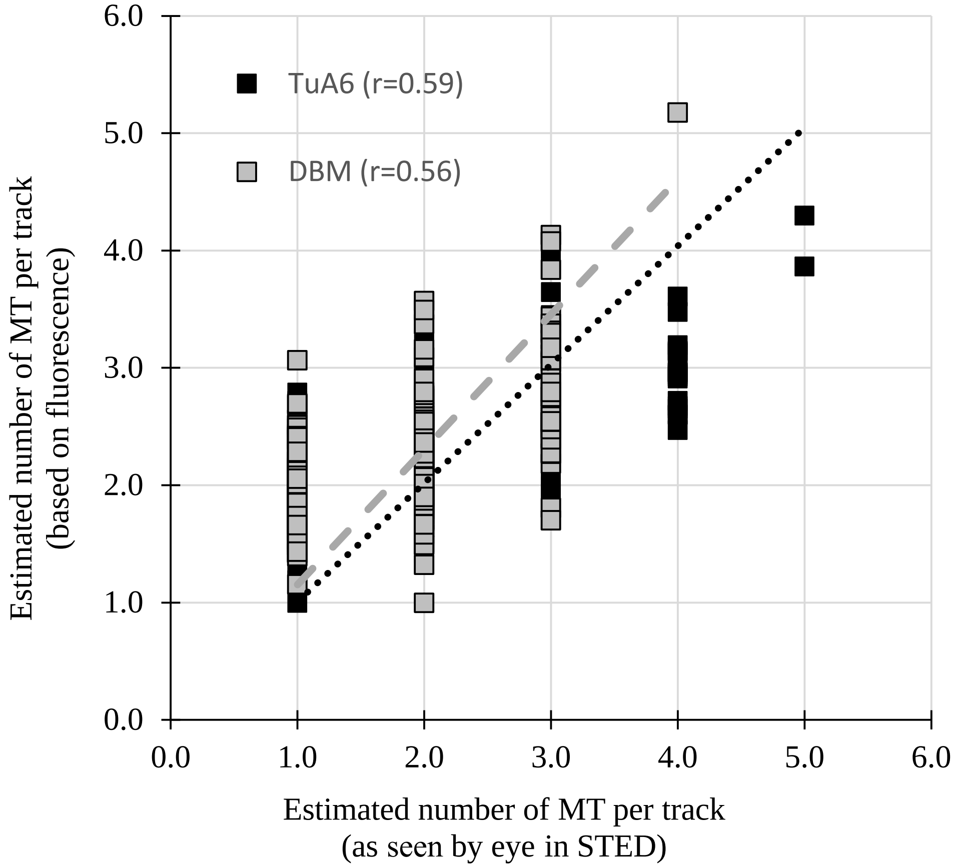

Figure S6: Linear correlation between the number of microtubules per track in confocal and in STED.

For each ROI analyzed we have an image in confocal mode and in STED super-resolution mode. The number of microtubule in each tracks has been estimated using the fluorescent signal from the non super-resolved image (Figure 2, S4 and S5). Here we compare that estimation to the estimated number of MT per track seen in the corresponding STED image.

For both genetic background the distributions show a statistically significant correlation between the two variables, p-value < 0.0001 according to a Spearman correlation for both genetic backgrounds.

Pearson's coefficients are also indicated on the graph.

Biology Open • Supplementary information

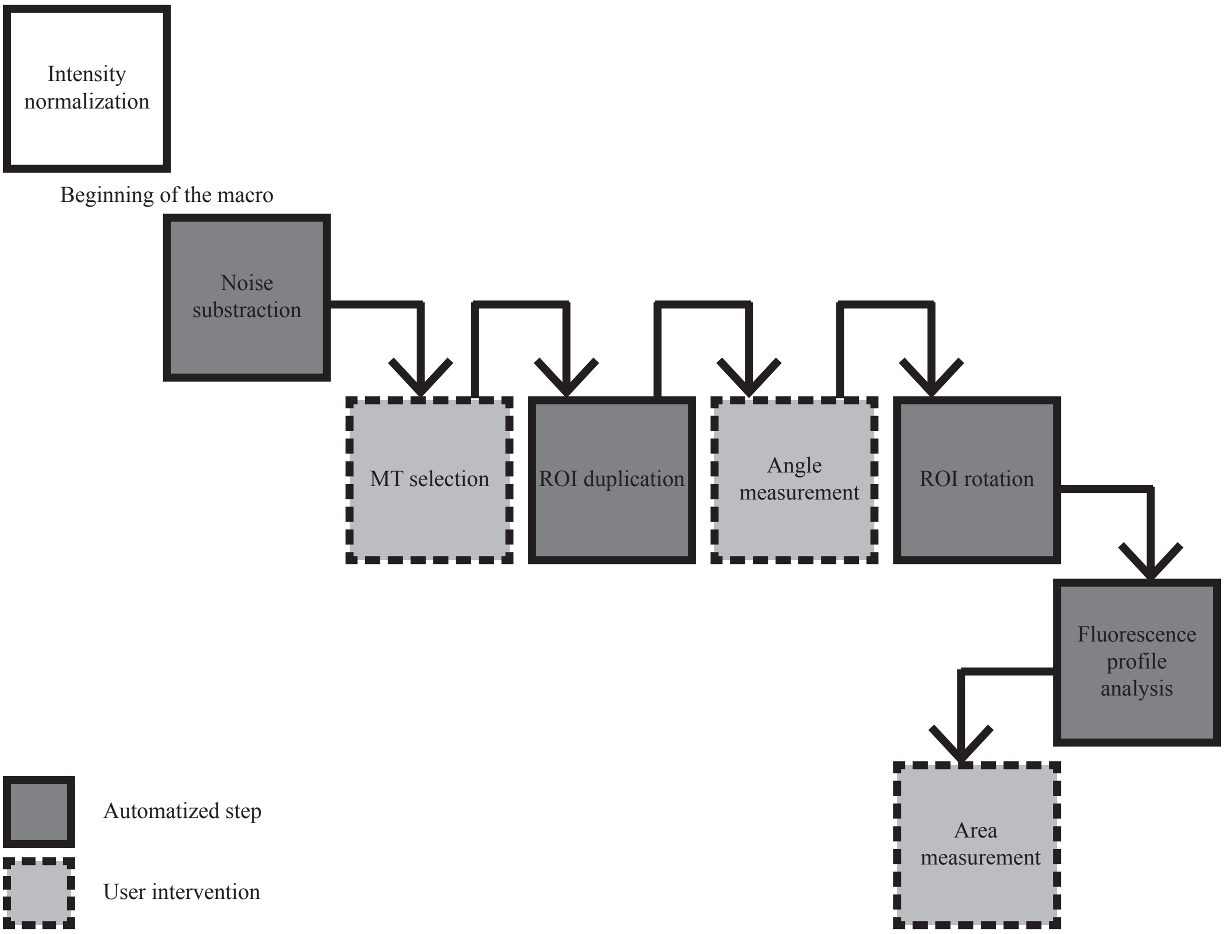

Figure S7: Workflow of the semi-automated approach designed for bundling quantification.

Biology Open • Supplementary information

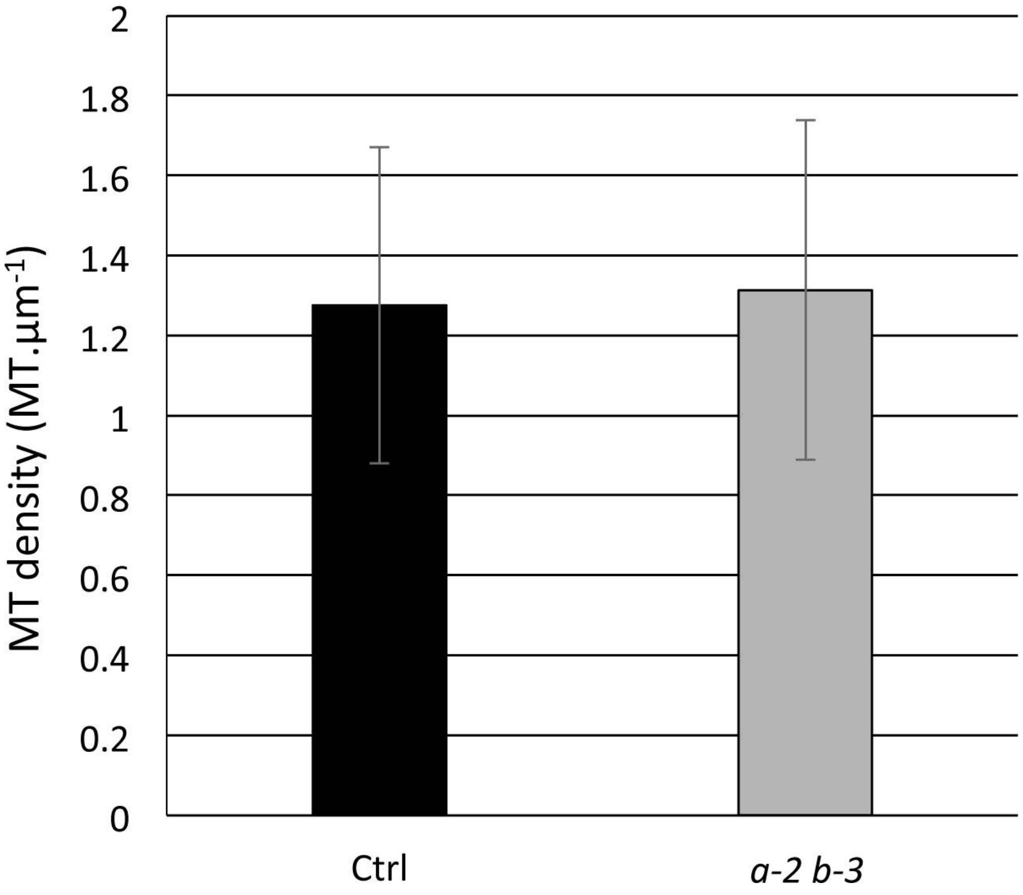

Figure S8: Density of microtubules per unit of length.

The histogram shows microtubule density per unit of length (µm) in the STED images illustrated in Figure 2. Cells present 1.27 ± 0.39 MT.μm^{-1} in the control and 1.31 ± 0.42 MT.μm^{-1} in the *eb1a-2 eb1b-3* double mutant (mean ± standard deviation to the mean).

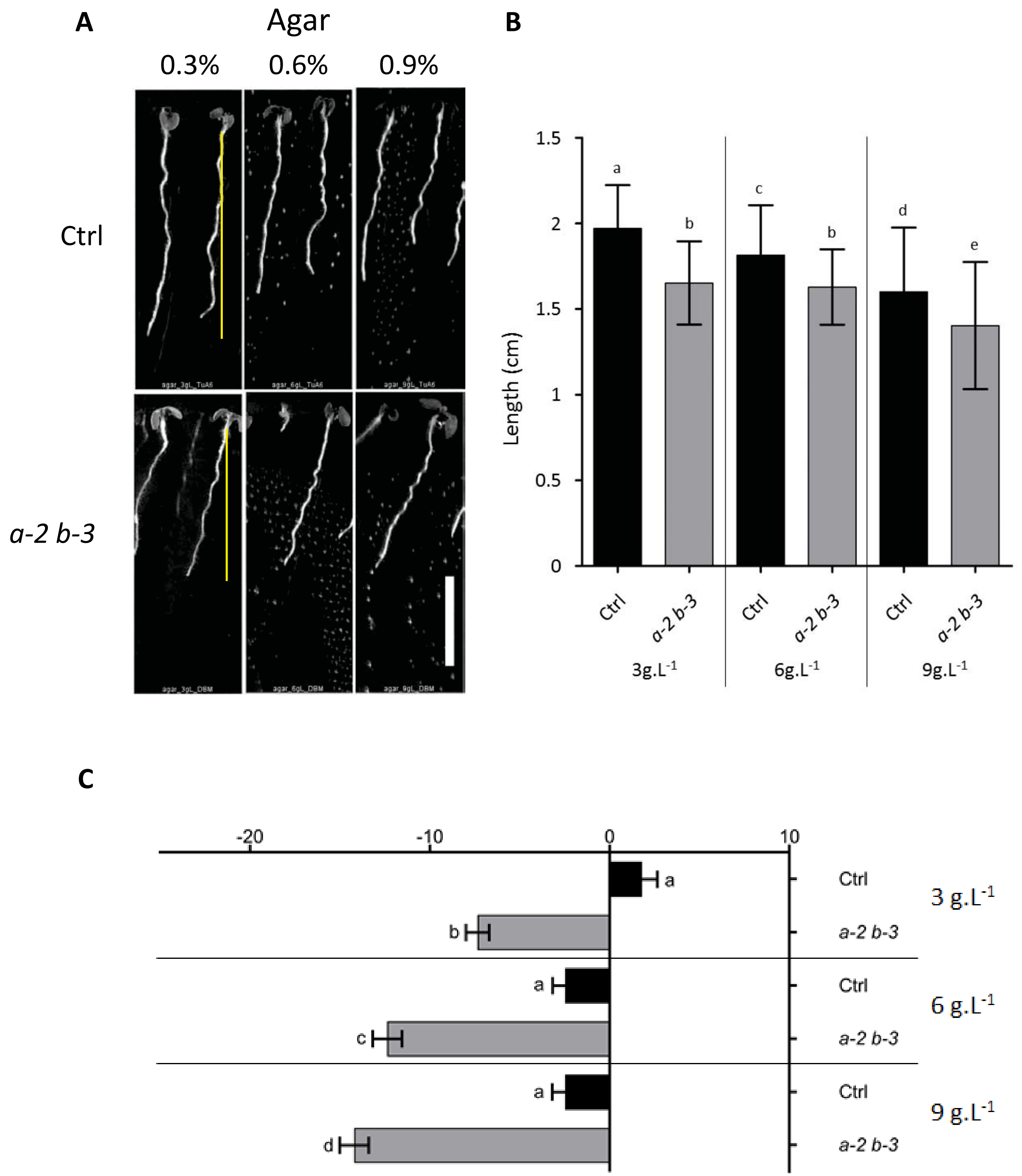
A
Agar
0.3%
0.6%
0.9%
Ctrl
a-2 b-3
B
Length (cm)
Ctrl
a-2 b-3
3g.L-1
6g.L-1
9g.L-1
C
-20
-10
0
10
Ctrl
a-2 b-3
3 g.L-1
6 g.L-1
9 g.L-1

Figure S9

Figure S9: Hypersensitivity of *eb1a-2 eb1b-3* double mutant roots to touch stimulation induced by medium hardness.

(**A**) Typical pictures illustrating the increase of root skewing and shortening as a function of the medium hardness for control and *eb1a-2 eb1b-3* double-mutant plants (both expressing 35S::GFP:TuA6). Scale bar, 1 cm. (**B**) Measurement of the root length as a function of the medium hardness (3/6/9 g.L^{-1} Agar containing medium). n=93/90/98 for the control and n=76/95/86 for the double mutant. Letters represent statistically significant differences (t-test, α=0.01). Bars indicate SEM. (**C**) Measurement of the root-skewing angle as a function of the medium hardness (3/6/9 g.L^{-1} Agar containing medium). Positive and negative angle represent rightward and leftward deviation respectively. n=89/96/103 for the control and n=84/91/90 for the double mutant. Letters represent statistically significant differences (t-test, α=0.01). Bars indicate SEM.

Biology Open • Supplementary information

Acronym	Line	Type of mutation	Ecotype	Source
a-2	*eb1a-2*	tDNA	Columbia	(Komaki et al., 2010)
b-3	*eb1b-3*	SNP	Columbia	(Komaki et al., 2010)
a-2 b-3	*eb1a-2 eb1b-3* 35S::GFP::TuA6	tDNA P	Columbia	(Komaki et al., 2010)
Control	35S::GFP::TuA6	tDNA	Columbia	(Ueda et al., 1999)

Table SI: Recap chart of plant lines used in the present study

Biology Open • Supplementary information

	**Ctrl**	***a-2 b-3***
**V_g (µm.min^{-1})**	5.25 (1.42)<br>n = 158	5.61 (1.63)<br>n = 125
**V_s (µm.min^{-1})**	-16.48 (8.05)<br>n = 93	-16.02 (12.30)<br>n = 64
**F_{cat} (min^{-1})**	0.776<br>n = 93	0.659<br>n = 64
**F_{res} (min^{-1})**	1.152<br>n = 20	0.928<br>n = 13
**Growth time (sec)**	7725.8	5825.5
**Growth length (µm)**	675.55	545.15
**Shrinkage time (sec)**	1041.4	840.17
**Shrinkage length (µm)**	286.08	224.30
**MT number**	158	125
**Plant number**	8	8

Table SII: Parameters of microtubule dynamic instability in control and *eb1a-2 eb1b-3* double mutant plants (both expressing 35S::GFP::TuA6). Values have been cumulated here from all measured trajectories, whereas Figure   indicates averaged values from 8 plants.
