# Supplementary material for: EB1 contributes to microtubule bundling and organization, along with root growth, in Arabidopsis thaliana
Source: Biol Open. 2018 Jun 26;7(8):bio030510. doi: 10.1242/bio.030510 (PMC6124560; doi:10.1242/bio.030510)
Supplement: First Person interview [file biolopen-7-030510-s2.pdf]

© 2018. Published by The Company of Biologists Ltd | Biology Open (2018): doi:10.1242/bio.030510: Supplementary information

FIRST PERSON

First person – Arthur Molines

First Person is a series of interviews with the first authors of a selection of papers published in Biology Open, helping early-career researchers promote themselves alongside their papers. Arthur Molines is first author on 'EB1 contributes to microtubule bundling and organization, along with root growth, in *Arabidopsis thaliana*', published in BiO. Arthur conducted the research in this article while a PhD student in the lab of Frederic Coquelle at Orsay University, Gif-sur-yvette, France. He is now a postdoc in Fred Chang's lab at the University of California, San Francisco, USA, investigating the interplay between cytoplasm biophysical properties and cytoskeleton dynamics.

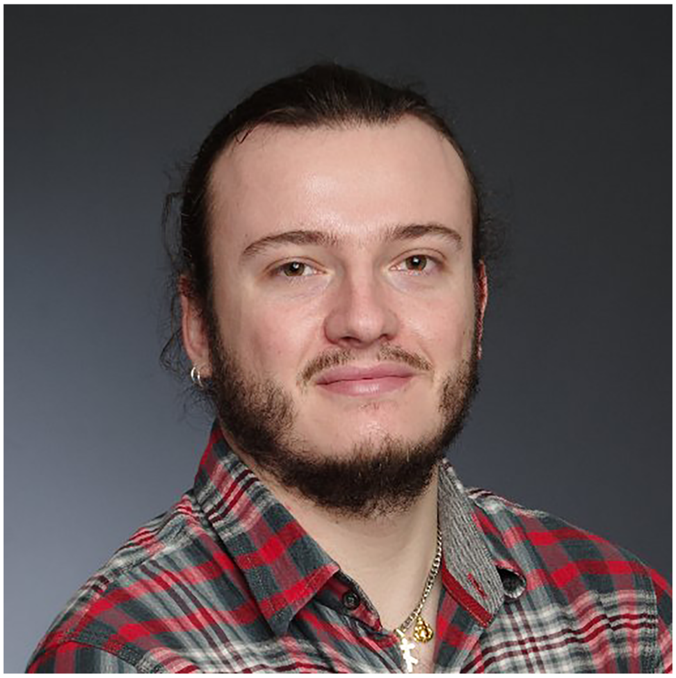

Arthur Molines

What is your scientific background and the general focus of your lab?

My academic training is in cellular biology and physiology, and my research background focuses on cytoskeleton dynamics and organization. My PhD project investigated the role of microtubule organization in plant growth and development. The lab where I did this work is paired with a microscopy facility, and focuses on making new imaging techniques available to the community and uses these methods to answer fundamental questions at the cellular or sub-cellular level in a variety of organisms. It was in this environment that I obtained my strong background in microscopy and image analysis. As a postdoc, I then moved to another model organism, fission yeast, to work on the interplay between cytoskeleton dynamic and cytoplasm biophysical properties. This new project and lab allow me to expand my background in cell biology and biophysics.

How would you explain the main findings of your paper to non-scientific family and friends?

Cells, the self-replicating biological machines that constitute living organisms, have to move and change their shapes to accomplish their functions. These movements and changes in shape are possible thanks to a network of fibers within cells called the cytoskeleton. The cytoskeleton is made of different fibers, among which microtubules are the stiffest and are key players in the organization of the cells' interior. In plants, microtubules also participate in the formation of the cell wall, an extremely rigid structure surrounding the cell that allows plants to rise against gravity. My work helps expand our understanding of the link between the organization of the microtubule network, the organization of the cell wall and the architecture of the whole plant. We have been able to correlate a defect in the organization of the microtubule network with changes in the cell wall and the root architecture.

What are the potential implications of these results for your field of research?

The main implications of my paper are a methodological achievement and a biologically relevant observation. We show that we can confidently estimate the number of microtubules per bundle using confocal microscopy and image analysis, and that a change in bundle abundance correlates with other macroscopic and sub-cellular phenotypes. The mechanism of formation and the biological functions of microtubule bundles have been open questions since these structures were first observed in the 1960s. These questions are still relevant today to fully understand the roles of the microtubule network in various fundamental cell functions such as mitosis, organelle movement (specifically in neurons), and mechanotransduction. The method developed in this paper might help to answer those questions.

> "The ability to generate large amounts of data and to analyze it in such detail is a dream come true for cell biologists."

What, in your opinion, are some of the greatest achievements in your field and how has this influenced your research?

Being a trained microscopist and relying a lot on image analysis for my research I'm not going to be very original here. Of course, I think that the latest combined developments in microscopy and image analysis are one of the greatest achievements that has influenced cell biology in recent years, pushing people forward to look at their samples with previously unachieved resolution in space and time. I have been particularly amazed by recent work that looks at organelle interactions in space and time, and that we now have the capability to quantify these interactions. The ability to generate large amounts of data and to analyze it in such detail is a dream come true for cell biologists.

Arthur Molines' contact details: Department of Cell and Tissue Biology, UCSF, San Francisco, CA 94143–0512, USA.

E-mail: a.t.molines@gmail.com; arthur.molines@ucsf.edu

Biology Open (2018): doi:10.1242/bio.030510: Supplementary information

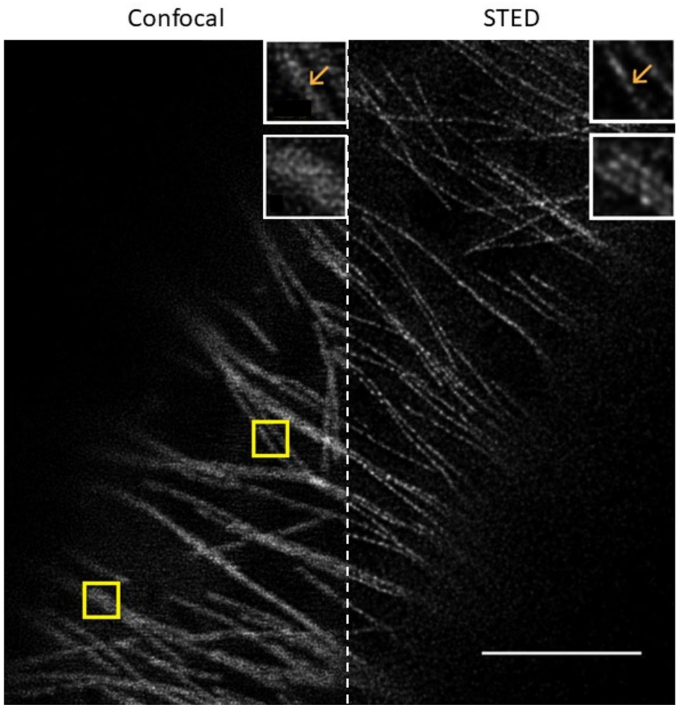

Arabidopsis thaliana epidermal cell expressing GFP-labeled tubulin in standard confocal (left) and in STED super-resolution (right).

What changes do you think could improve the professional lives of early-career scientists?

For most of us, research is a passion and because of it, we are willing to make a lot of sacrifices to be able to continue our passion. These sacrifices include, but are not limited to, having to live with a not-so-high level of income during grad school and most of our postdoc and then having to move, sometimes to the other side of the globe, to pursue our career. Increasing incomes for grad-school students and postdocs would decrease the financial pressure and allow people to have more freedom to do activities outside the lab and find their work-life balance, making them happier and more efficient. Mentorship from early-career scientists that are one step ahead of you and senior scientists would be a great tool to completely understand what is expected from us at each career step.

What's next for you?

Since obtaining my PhD in France I have moved to the USA (University of California, San Francisco) for a postdoctoral position on a new project. Through this project, I plan on expanding my knowledge of cytoskeleton and cellular biophysics to pursue a career in academia.

Reference

Molines, A. T., Marion, J., Chabout, S., Besse, L., Dompierre, J. P., Mouille, G. and Coquelle F. M. (2018). EB1 contributes to microtubule bundling and organization, along with root growth, in *Arabidopsis thaliana*. *Biol. Open* **7**: bio030510.

Biology Open
